# Effectiveness of non-invasive respiratory support strategies in patients with COVID-19: A systematic review and meta analysis

**DOI:** 10.1016/j.amsu.2022.104827

**Published:** 2022-11-08

**Authors:** Vinesh Kumar, Umair Arshad Malik, Reshman Kumari, Berkha, Vijay Kumar, Manoj Kumar, Simran, Suman, Bushra Nasim

**Affiliations:** a Department of Medicine, Chandka Medical College, Larkhana, Pakistan; b Department of Medicine, Aga Khan University, Karachi, Pakistan; c Department of Medicine, Peoples University of Medical & Health Sciences for Women, Nawabshah, Pakistan; d Department of Medicine, Liaquat University of Medical & Health Sciences, Jamshoro, Pakistan; e Department of Medicine, Jinnah Sindh Medical University, Karachi, Pakistan; f Department of Medicine, Dow Medical College, Karachi, Pakistan

**Keywords:** Continuous positive airway pressure, High flow nasal oxygen, Tracheal intubation

## Abstract

**Background:**

BackgroundThe effectiveness of non-invasive respiratory strategies, namely CPAP and HFNO, in reducing the risk of mortality and tracheal intubation in patients with severe COVID-19 is not well established.

**Methods:**

A thorough literature search was conducted across 3 electronic databases (Medline, EMBASE and Cochrane Central) from inception through July 2022. Randomized controlled trials (RCTs) and observational studies assessing the impact of CPAP or HFNO on clinical outcomes in patients infected with COVID-19 were considered for inclusion. End-points included all-cause mortality and risk of tracheal intubation. Evaluations were reported as risk ratios (RRs) with 95% confidence intervals (CI) and analysis was performed using a random effects model. I^2^ index was used to assess heterogeneity.

**Results:**

From the 1041 articles retrieved from initial search, 7 potentially relevant studies (n = 2831 patients) were included in the final analysis. Compared to conventional oxygen therapy, non-invasive respiratory strategies reduced the risk of tracheal intubation (RR = 0.84, [95% CI 0.72, 0.98]; p = 0.02, I^2^ = 43%) and all-cause mortality (RR = 0.83, [95% CI 0.71–0.97]; p = 0.02, I^2^ = 0%) in patients infected with COVID-19 However, reduction in length of hospital stay was not significant between the non-invasive respiratory group and conventional oxygen therapy (MD = −0.60, [95% CI -2.17 – 0.98]; p = 0.46, I^2^ = 26%).

**Conclusion:**

This meta-analysis supports the application of non-invasive respiratory strategy is feasible as it can delay the start of tracheal intubation and reduce mortality rates among patients infected with COVID-19.

## Highlights

• Data indicate that high-flow oxygen treatment may reduce the requirement for endotracheal intubation but not death rates.
• Significant variation exists in guidelines regarding the use of non-invasive respiratory strategies in COVID-19 patients.
• Randomized controlled trials to assess the benefits of non-invasive respiratory support strategies among COVID-19 patients.

## 1 Introduction

Arterial hypoxemia is a primary characteristic of severe patients infected with coronavirus (COVID-19) [1]. In the early stages of the COVID-19 pandemic, worldwide experiences showed the potential for intensive care units (ICUs) may become overrun. A significant fatality rate was seen among patients requiring invasive mechanical ventilation. This necessitated identifying ways to lessen the necessity for intrusive mechanical ventilation as an urgent matter of public health. Oxygen supplementation is considered in its management to increase oxygenation and support respiratory effort using a variety of support modalities [2]. Evidence from recent observational studies indicates that patients who undergo invasive mechanical ventilation and early tracheal intubation are associated with a relatively higher risk of mortality [3,4]. Hence, several researchers have issued warnings regarding early intubation and mechanical ventilation during the COVID-19 epidemic [5]. However, evidence also shows that in non-intubated patients with acute respiratory failure, transpulmonary pressure swings during spontaneous breathing and labored respiratory drives can also result in patient-self-inflicted lung damage [6], supporting the use of mechanical ventilation in clinical settings.

In recent years, non-invasive respiratory support (NRS) strategies such as continuous positive airway pressure (CPAP) and high-flow nasal oxygen (HFNO) have shown to be potentially appealing alternatives to avoid early intubation and invasive mechanical ventilation [[7], [8], [9]]. Non-invasive CPAP ventilation support involves the use of either helmets, or different kinds of tight masks that provide an inspired fraction of oxygen (FiO2) of 100%. High-flow nasal oxygen (HFNO2) therapy is usually applied via a wide-bore nasal cannula. It provides up to 60L/min of a heated and humified gas mixtures (at an adjustable mix medical of oxygen and room air**).**

Data indicate that high-flow oxygen treatment may reduce the requirement for endotracheal intubation and the likelihood of therapy progression in patients with acute hypoxemic respiratory failure but does not influence death rates [10]. While worldwide guidelines and early observational studies suggested utilizing high-flow oxygen treatment through a nasal cannula to initially treat patients with severe COVID-19, there is very limited data to support this [11]. There is no conclusive evidence supporting the use of NRS strategies in avoiding adverse events in patients with COVID-19. Moreover, significant variation exists in international recommendations and clinical practice because of the lack of data supporting the use of NRS in COVID-19 patients [5,12]. On this basis, there was a need for a meta-analysis to determine whether CPAP or HFNO, compared with conventional oxygen therapy, reduces the need for tracheal intubation or mortality in patients with acute hypoxemic respiratory failure due to COVID-19. Therefore, we conducted a meta-analysis to evaluate the risk of tracheal intubation and mortality among COVID-19 patients receiving NRS compared with those on conventional oxygen therapy.

## 2 Methods

This meta-analysis was conducted in accordance with Preferred Reporting Items for Systematic review and Meta-Analyses (PRISMA) guidelines [13] and The MeaSurement Tool to Assess systematic Review (AMSTAR 2) [14]. This study was registered in National Institute for Health Research (NIHR) International prospective register of systematic reviews (PROSPERO) (Identification No. CRD42022349601) [15].

## 3 Data sources and search strategy

Two reviewers (VK and RK) independently searched PubMed, Cochrane CENTRAL, and Embase databases through 20^th^ July 2022 using the following terminologies and their MESH terms: “high flow nasal oxygen” “HFNO”, “high flow nasal canula”, “HFNC”, “continuous positive airway pressure”, “CPAP”, and “non-invasive respiratory support”. We also reviewed other data sources: bibliographies of editorials and relevant reviews from major medical journals, conference proceedings for indexed abstracts, and databases of grey/unpublished literature. The detailed search strategy for both databases is provided in Supplemental Table S1.

## 4 Study selection

Studies were included if they were: (a) randomized controlled trials (RCTs) or observational studies; (b) compared the outcomes between NRS and conventional oxygen therapy; and (c) included patients with positive real-time Reverse Transcriptase- Polymerase Chain Reaction (RT-PCR) assays for COVID-19 infection. In case of any discrepancy third reviewer (UAM) was consulted. In all the included studies, NRS was continued until the patient stabilized or reached one of terminal endpoints i.e., need for tracheal intubation or mortality. All studies retrieved were compiled in Endnote Reference Library (Version X7.5; Clarivate Analytics, Philadelphia, Pennsylvania) software, where the duplicates were identified and removed. All the remaining articles were then thoroughly reviewed to ensure that they met our predefined eligibility criteria.

## 5 Data extraction and quality assessment

Two reviewers (VK and RK) extracted outcomes of interest that included the incidence of tracheal intubation, all-cause mortality, and length of hospital stay. Risk ratios (RRs) with 95% CIs, pooled using Mantel–Haenszel weighted random-effects model. The pooled analyses were visually represented with forest plots. All analyses were conducted on Review Manager (Version 5.4; Cochrane Collaboration). The Cochrane Risk of Bias Tool (CRBT) for RCTs and the New Castle Ottawa Scale (NOS) for observational studies were used to assess the risk of biases. Other study characteristics like the number of participants, publication year, length of follow-up, and mean/median ages were also extracted.

## 6 Statistical analysis

Meta-analysis was performed using RevMan (version 5.3; Copenhagen: The Nordic Cochrane Centre, The Cochrane Collaboration). Outcomes of interest were presented as RRs with 95% CIs and were pooled using an inverse variance weighted random-effects model. The pooled analyses were visually represented with forest plots. Higgins I^2^ was used to evaluate heterogeneity across studies. A value of 25–50% was deemed mild, 50–75% moderate, and >75% severe. Publication bias was assessed using a funnel plot for the outcome of Tracheal Intubation. A P-value of less than 0.05 was considered significant in all cases.

## 7 Results

### 7.1 Literature search results

**Fig. 1:**
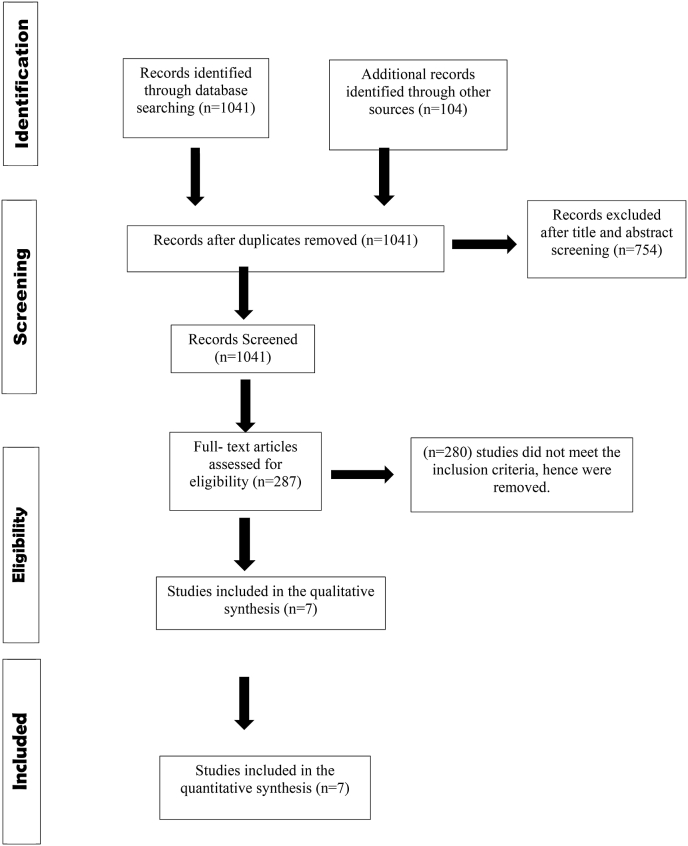
PRISMA Flowchart summarizing the literature search process.

Six studies (4 RCTs and 2 observational studies) containing 2831 patients (1506 in the non-invasive respiratory group and 1325 in the control group) met our inclusion criteria and were included in this meta-analysis [10,[16], [17], [18], [19]]. The PRISMA flowchart summarizing the literature search process is provided in Fig. 1.

### 7.2 Study characteristics and quality assessment

The baseline characteristics and the risk of bias assessment of the included studies are presented in Table 1. The mean age of patients ranged from 51 to 71 years. The percentage of males varied from 60.9 to 69.2% between studies. Observational studies were assessed to be of moderate to high quality, achieving scores from 6 to 7 out of a maximum of 9 on the Newcastle Ottawa scale (Supplemental Table 1). Similarly, RCTs were deemed to be of generally low risk of bias according to the CRBT (Supplemental Fig. S1). The publication bias can be visualized by the funnel plot (Supplemental Fig. S2)

**Table 1:** Characteristics of included studies and risk of bias assessment.

Study, Country	Year	Study Design	Total Patients (N)	Gender Distribution (Male)	Age (Year)	Number of Patients (N)	
						In Treatment Arm	In Control Arm
RECOVERY-RS	2022	RCT	1273	844	57	792	724
HiFLo-Covid	2021	RCT	220	134	60	109	111
Walker et al.	2020	Retrospective Cohort	294	183	71	45	249
Nair et al.	2021	RCT	109	NA	NA	55	54
Franco et al.	2020	Retrospective Cohort	670	464	68	493	177
Teng et al.	2021	RCT	22	15	55	12	10

RECOVERY-RS: Respiratory Support: Respiratory Strategies in patients with coronavirus COVID-19 – CPAP, high-flow nasal oxygen, and standard care; HiFLo-COVID: High-Flow Nasal Cannula in Severe COVID-19 With Acute Hypoxemic Respiratory Failure; NA: Not available.

## 8 Outcome analysis

### 8.1 Tracheal Intubation

**Fig. 2:**
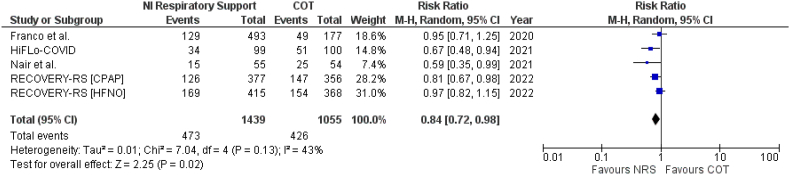
Forest plots for the effect of Non-invasive Respiratory Support (NRS) on Incidence of Tracheal Intubation, in patients with COVID-19.

Incidence of tracheal Intubation [Fig. 2]: Five studies (4 RCTs, 1 observational) reported the incidence of tracheal Intubation. Our pooled analysis shows that the incidence of tracheal intubation is significantly lower in COVID-19 patients on NRS compared with those on conventional oxygen therapy (RR = 0.84, [95% CI 0.72, 0.98]; p = 0.02, I^2^ = 43%).

### 8.2 All-cause mortality

**Fig. 3:**
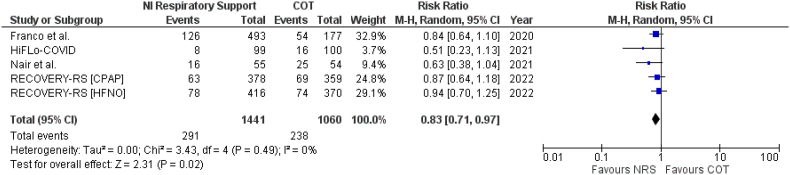
Forest plots for the effect of Non-invasive Respiratory Support (NRS) on Mortality, in patients with COVID-19.

Incidence of all-cause mortality [Fig. 3]: Five studies (4 RCTs, 1 observational) reported the outcome incidence of all-cause mortality. A significant reduction in the incidence of all-cause mortality was noted among patients undergoing NRS (RR = 0.83, [95% CI 0.71–0.97]; p = 0.02, I^2^ = 0%).

### 8.3 Length of hospitalization (in days)

**Fig. 4:**
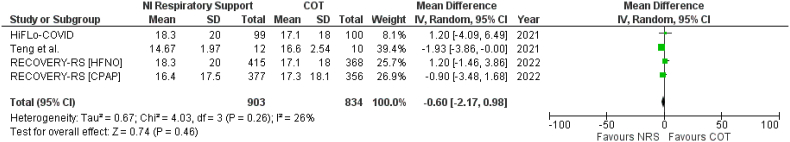
Forest plots for the effect of Non-invasive Respiratory Support (NRS) on Length of Hospital Stay, in patients with COVID-19.

Reduction in length of hospital stay [Fig. 4]: Four studies (4 RCTs) reported the outcome reduction in length of hospital stay. Our pooled analysis shows that reduction in length of hospital stay was not significant between the NRS group and conventional oxygen therapy (MD = −0.60, [95% CI -2.17 – 0.98]; p = 0.46, I^2^ = 26%)

## 9 Discussion

Our meta-analysis suggests that patients infected with COVID-19 undergoing NRS are significantly less likely to undergo tracheal intubation. Similarly, there was a significant association between NRS and reduced risk of all-cause mortality. However, there was no significant difference between NRS strategies and conventional oxygen therapy concerning the length of hospital stay. These findings support the early use of CPAP and HFNO strategies in patients infected with severe COVID-19.

Previous trials evaluating the effectiveness of HFNO and CPAP on the risk of tracheal intubation and mortality in patients with COVID-19 showed that NRS strategies reduce the risk of tracheal intubation but not. For example, The RECOVERY-RS study indicated that CPAP significantly reduced tracheal intubation but not death; nonetheless, the broad 95% confidence interval limited reaching a definitive conclusion regarding the effect on mortality in this study [10]. In the High-Flow Nasal Cannula in Severe COVID-19 With Acute Hypoxemic Respiratory Failure (HiFLo-Covid) trial, HFNO was compared with standard oxygen treatment in 220 persons with severe COVID-19 in three hospitals in Colombia. HFNO decreased the requirement for tracheal intubation (hazard ratio, 0.62; 95% confidence interval, 0.39–0.96) and time to clinical recovery, but no significant reduction in mortality was seen [16]. In contrast, among patients with acute respiratory failure, a recent systematic review and meta-analysis of 25 randomized clinical trials conducted before the COVID-19 pandemic (3,804 patients) evaluated the therapeutic benefit of non-invasive ventilation (with or without pressure support) and HFNO compared to traditional oxygen treatment show that non-invasive ventilation through a face mask was significantly related with a decreased risk of death and tracheal intubation across 14 studies including 1275 participants [20].

The current meta-analysis establishes that the use of HFNO and CPAP reduces both the risk of tracheal intubation and mortality among patients with acute respiratory failure due to COVID-19. This is analogous to small individual trials ascertaining the risk of mortality and tracheal intubation among patients with acute hypoxemic respiratory failure due to COVID-19. None of the studies have established a significant association between reduced mortality risk and the use of an NRS strategy. This could be because previous studies were limited by decreased event rates due to early study terminations and fewer participants enrolled. However, pooling data from all these studies showed that NRS has the potential mortality benefit as well, further supporting its administration in patients infected with COVID-19.

NRS provided at early stages can offer theoretical advantages over conventional oxygen therapies, including improvements in the inspiratory effort, minute volume, respiratory rate, lung volumes, dynamic lung compliance, transpulmonary pressure, and lung homogeneity [21]. Preventing tracheal intubation in patients infected with COVID-19 can help optimize resources during the pandemic and reduce complications related to invasive mechanical ventilation, such as sedation, delirium, and neuromuscular paralysis [22]. Patients receiving NRS demonstrate a quicker clinical recovery. However, the underlying mechanisms are less clear than those implicated in avoiding intubation. Theoretically, very early relaxation of inspiratory effort could decrease self-inflicted lung injury, which should affect clinical results [23]. Further evidence-based studies are required to fully understand the underlying mechanisms that determine the efficacy and devise a modified protocolized management plan to optimize the treatment of patients with COVID-19 pneumonitis.

Alternative non-invasive respiratory support techniques may be more effective than CPAP. In a prior randomized controlled trial, CPAP with helmets was found to be more effective than CPAP with face masks at reducing the need for endotracheal intubation (62% vs. 18%). It has also been demonstrated that high-flow oxygen administered using a nasal cannula is beneficial in treating acute hypoxemic respiratory failure. However, due to the substantial oxygen consumption required by this technique, its usage is limited. Indirect ventilation using a helmet (with pressure support) vs. HFNO was directly compared between the two non-invasive respiratory methods in the Helmet Noninvasive Ventilation Versus High-Flow Oxygen Therapy in Acute Hypoxemic Respiratory Failure (HENIVOT) trial [24]. There was no significant difference in the primary outcome of days free of respiratory support in 110 COVID-19 patients enrolled across 4 ICUs; however, considerably fewer patients in the helmet non-invasive ventilation group required tracheal intubation (odds ratio, 0.41; 95% CI, 0.18–0.89). Further study is necessary to better understand the increased infectious risk that non-invasive breathing techniques like CPAP provide to healthcare professionals. Currently, randomized controlled trials are being conducted to determine the best non-invasive pressure support technique for minimizing the necessity for intubation.

Several limitations in this study should be noted. This is the study-level meta-analysis as patient-level data were not available. The lower tracheal intubation rate in the NRS group may be driven by greater willingness among clinicians to delay tracheal intubation in this group. In addition, we could not separately evaluate the effectiveness of CPAP and HFNO because of the lack of data available on each strategy.

## 10 Conclusion

To conclude, this is the first meta-analysis showing that the application of NRS is feasible as it can unduly delay the start of tracheal intubation and reduce mortality rates among patients infected with COVID-19 pneumonitis.

## Ethical approval

Ethics committee approval was not required because manuscript uses publicly available data.

## Sources of funding

None to Declare.

## Author contribution

Vinesh Kumar and Manoj Kumar conceived the idea and designed the study, Umair Arshad Malik and Berkha collected the data and analysed it, Reshman Kumari, and Vijay Kumar drafted the manuscript, Simran and Suman conducted literature search and created the illustrations, Bushra Nasim revised the manuscript critically.

## Registration of research studies

Name of the registry: National Institute for Health Research (NIHR)

International prospective register of systematic reviews (PROSPERO)

Unique Identifying number or registration ID: CRD42022349601.

Hyperlink to your specific registration (must be publicly accessible and will be checked): https://www.crd.york.ac.uk/PROSPERO/display_record.php?RecordID=349601.

## Guarantor

Umair Arshad Malik.

## Provenance and peer review

Not commissioned, externally peer reviewed.

## Consent

NA.

## Declaration of competing interest

None to Declare.
