# Supplementary material for: Effectiveness of non-invasive respiratory support strategies in patients with COVID-19: A systematic review and meta analysis
Source: Ann Med Surg (Lond). 2022 Nov 8;84:104827. doi: 10.1016/j.amsu.2022.104827 (PMC9640384; doi:10.1016/j.amsu.2022.104827)

**Supplementary Materials**

**Supplemental Figure 1. Risk of Bias of RCTs**


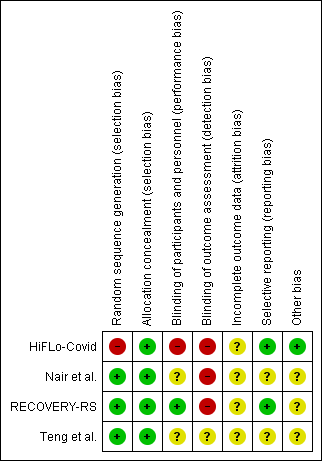


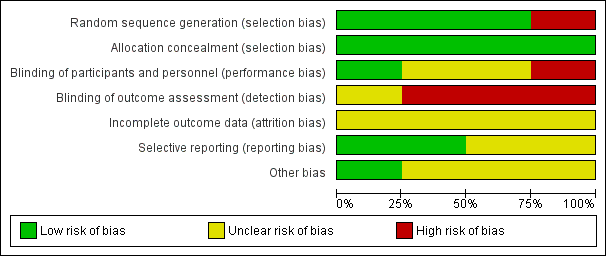


^1^ Risk of bias for randomized controlled trials includes seven domains: random sequence generation (selection bias), allocation concealment (selection bias), blinding of participants and personnel (performance bias), blinding of outcome assessment (detection bias), incomplete data reporting (attrition bias), selective reporting (reporting bias), and other bias. Each domain includes one *, and ≥ 5 * indicates good trial quality

**Supplemental Figure 2. Funnel Plot Showing no Publication Bias in Studies Included for the** **outcome of Tracheal Intubation.**


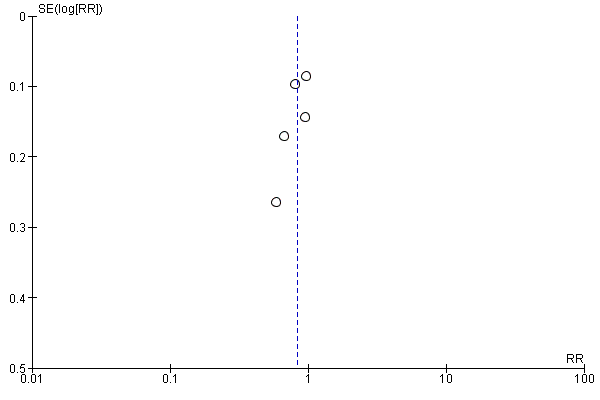

Supplement: Multimedia component 3 [file mmc3.docx]
